# What Lies Behind Successful Regulation? A Qualitative Evaluation of Pilot Implementation of Kenya’s Health Facility Inspection Reforms

**DOI:** 10.34172/ijhpm.2021.90

**Published:** 2021-08-25

**Authors:** Eric Tama, Irene Khayoni, Catherine Goodman, Dosila Ogira, Timothy Chege, Njeri Gitau, Francis Wafula

**Affiliations:** ^1^Institute of Healthcare Management, Strathmore University Business School, Strathmore University, Nairobi, Kenya.; ^2^Department of Global Health and Development, London School of Hygiene and Tropical Medicine, University of London, London, UK.; ^3^World Bank Group, Nairobi, Kenya.

**Keywords:** Regulation, Inspection, Patient Safety, Private Sector, Kenya

## Abstract

**Background:** Health facility regulation in low- and middle-income countries (LMICs) is generally weak, with potentially serious consequences for safety and quality. Innovative regulatory reforms were piloted in three Kenyan counties including: a Joint Health Inspection Checklist (JHIC) synthesizing requirements across multiple regulatory agencies; increased inspection frequency; allocating facilities to compliance categories which determined warnings, sanctions and/or time to re-inspection; and public display of regulatory results. The reforms substantially increased inspection scores compared with control facilities. We developed lessons for future regulatory policy from this pilot by identifying key factors that facilitated or hindered its implementation.

**Methods:** We conducted a qualitative study to understand views and experiences of actors involved in the one-year pilot. We interviewed 77 purposively selected staff from the national, county and facility levels. Data were analyzed using the framework approach, identifying facilitating/hindering factors at the facility, inspection system, and health system levels.

**Results:** The joint health inspections (JHIs) were generally viewed as fair, objective and transparent, which enhanced their perceived legitimacy. Interactions with inspectors were described as friendly and supportive, in contrast to the punitive culture of previous inspections when bribery had been common. Inspector training and use of an electronic checklist were strongly praised. However, practical challenges with transport, route planning and budgets highlighted the critical nature of strong logistical management. The effectiveness of inspection in improving compliance was hampered by limitations in related systems, particularly facility licensing, enforcement of closures and, in the public sector, control of funds. However, an inclusive reform development process had led to high buy-in across regulatory agencies which was key to the system’s success.

**Conclusion:** Effective facility inspection involves more than "hardware" such as checklists, protocols and training. Cultural, relational and institutional "software" are also crucial for legitimacy, feasibility of implementation and enforceability, and should be carefully integrated into regulatory reforms.

## Background

**Key Messages**

** Implications for policy makers**

- Effective facility inspection involves much more than the “hardware” elements, such as checklists, protocols and training.
- Relational and cultural “software” are also crucial for legitimacy, feasibility and enforceability, and should be carefully integrated into regulatory reforms.
- During the inspection reforms piloted in Kenya key software elements included (*i*) enhancing perceptions of objectivity, fairness and transparency; (*ii*) an inspection culture supportive to facility staff; (*iii*) strong logistical planning and management; and (*iv*) high level buy-in across regulatory stakeholders.
- The impact of inspections is also heavily influenced by the related systems for enforcement, licensing and financing, all of which need to work well if compliance is to substantially improve.

** Implications for the public**

In a context of a large and growing private facility sector and a weak regulatory system, patient safety can easily be compromised. In Kenya a pilot of an innovative set of regulatory reforms has improved facility compliance. We identified key factors that facilitated or hindered the pilot’s success. We found that it is not enough to have the right “hardware” in terms of checklists, protocols and training. Successful inspection also required “software” elements such as perceived fairness and legitimacy, a supportive culture, strong planning and management, and high-level buy-in. These lessons are important for the design of successful regulatory reforms that can ensure health facilities comply with patient safety standards and improve quality of care.

Most low- and middle-income countries (LMICs) have pluralistic health systems, with coexistence of public and private provision and financing of healthcare.^1^ An analysis of World Health Survey data of 39 LMICs found that over 40% of outpatient visits were to either a private-for-profit or a faith based organization (FBO)/non-governmental organization (NGO) facility.^2^ Recent years have seen increased involvement of private facilities in providing health services, reflecting inadequate coverage and quality of the public sector, rapid urbanization, and increasing disposable incomes.^3^ These private facilities range from small clinics to large multi-specialty hospitals and hospital chains, with a mix of for-profit and not-for-profit orientation.^4^

 LMIC governments struggle to regulate these pluralistic and highly fragmented health systems effectively.^5^ Regulation can be broadly defined to encompass not only government rules, but also community accountability, contracting arrangements, and quality improvement or assurance activities.^6^ Here we focus on the narrower definition around “command and control” strategies, where the force of law is used to pursue policy objectives and guard public safety.^7^ These statutory regulations are outdated in many LMICs,^3^ and often focus on market entry, with limited effective control of operation post-entry.^8^ Governments often have incomplete information even on the size of the private sector, with unregistered facilities common.^6,9^ Regulatory systems in LMICs have also failed to keep pace with recent thinking in regulatory policy, such as “risk-based” and “responsive” regulation.^7^ Risk-based regulation involves prioritizing resources to regulatees expected to be highest risk, while responsive regulation is based on a pyramid of sanctions beginning with dialogue and persuasion, gradually escalating through warnings and repeat inspections, to more punitive measures (“soft words before hard”).^10^ Documentation of strategies aimed at strengthening regulation in LMICs is poor^11^ and their evaluation very limited except for a few randomised trials in pharmacies.^5,12^ The use of other approaches such as case studies and qualitative methods for rigorous investigation of regulatory implementation is also rare.^13,14^

 Compared to most other sub-Saharan Africa countries, Kenya’s private healthcare sector is relatively developed, yet remains poorly regulated.^15^ Over half (52.6%) of Kenya’s health facilities are private, of which 78% are for-profit, 16% FBO and 6% NGO.^16^ Although health service delivery is devolved to 47 semi-autonomous counties, regulation remains the responsibility of the national government, implemented by eight regulatory agencies (for doctors and dentists, clinical officers, nurses, public health officers (PHO), pharmacies, laboratories, radiologists and nutrition and dieticians), and overseen by the Ministry of Health (MoH).

 Kenya’s eight regulatory agencies were mandated to visit facilities independently and conduct inspections using their own criteria. However, it was estimated that less than 5% of private facilities received any inspection each year, and even where inspections were done, records were poorly kept, making follow-up action difficult. In addition, there were complaints of conflicting standards across regulators, and lack of clarity on sanctions for non-compliance.^17^ These weaknesses were reflected in poor regulatory compliance, with 98% of Kenyan facilities failing to meet minimum patient safety standards in a nationwide survey.^18^

###  The Joint Health Inspections Pilot

 It was against this background that the Kenyan MoH proposed an innovative joint facility inspection system that combined and refined standards from all eight regulatory agencies into one tool for inspecting public and private facilities. This Joint Health Inspection (JHI) system incorporated insights from risk-based and responsive regulation. Facilities were categorized into compliance categories based on their inspection score (Table 1). The lowest compliance/highest risk facilities were to be immediately closed, while for other facilities, time to reinspection was positively related to compliance category. Following the principles of responsive regulation, facilities outside the lowest compliance category were not penalized for infringements on their first inspection, but informed about their performance, with closure only to be implemented if sufficient improvements had not taken place at their third inspection.

**Table 1:** JHIC Scores and Follow-up Actions

**JHIC Score**	**Compliance Category**	**Follow-up Action**
≤10% or no license	Non-compliant	Immediate closure
11%-40%	Minimally compliant	Re-inspection in 3 months Facility will be closed if it does not score over 40% in the third inspection
41%-60%	Partially compliant	Re-inspection in 6 months Facility will be closed if it does not score over 60% score in the third inspection
61%-75%	Substantially compliant	Re-inspection in 12 months
>75%	Fully compliant	Re-inspection in 24 months

Abbreviation: JHIC, Joint Health Inspection Checklist. Source:^17^

 The MoH, with the support of the World Bank Group, piloted the JHI in 2017 through a randomised controlled trial (RCT) in the three counties of Kakamega, Kilifi and Meru, termed the Kenya Patient Safety Impact Evaluation (KePSIE). Market centres in each county were randomly assigned to one of three arms, with roughly 400 facilities per arm. In arms 1 and 2, all public and private facilities were inspected at least once annually following the JHI protocol. In addition, in arm 2, inspection performance scorecards were publicly displayed at the facility. Arm 3 continued with normal practice, which effectively meant no facilities were inspected.

 The trial was the largest to date on facility regulation in an LMIC setting. A wide range of stakeholders were involved in implementation. Advisory and governance support was provided by a World Bank core team and the KePSIE Task Force comprising national and county health officials, managers of the regulatory bodies and private sector representatives. A MoH coordinator was responsible for managing and supervising implementation, with inspections carried out by 8 nationally-seconded inspectors across the three counties. Inspectors were supported by two World Bank field staff in each county, who also oversaw the KePSIE evaluation. Closures of facilities or facility departments identified to lack valid licenses during inspections were implemented during a subsequent visit by MoH staff. A focal person was identified within each County Health Management Team to support the process.

**Figure 1:**
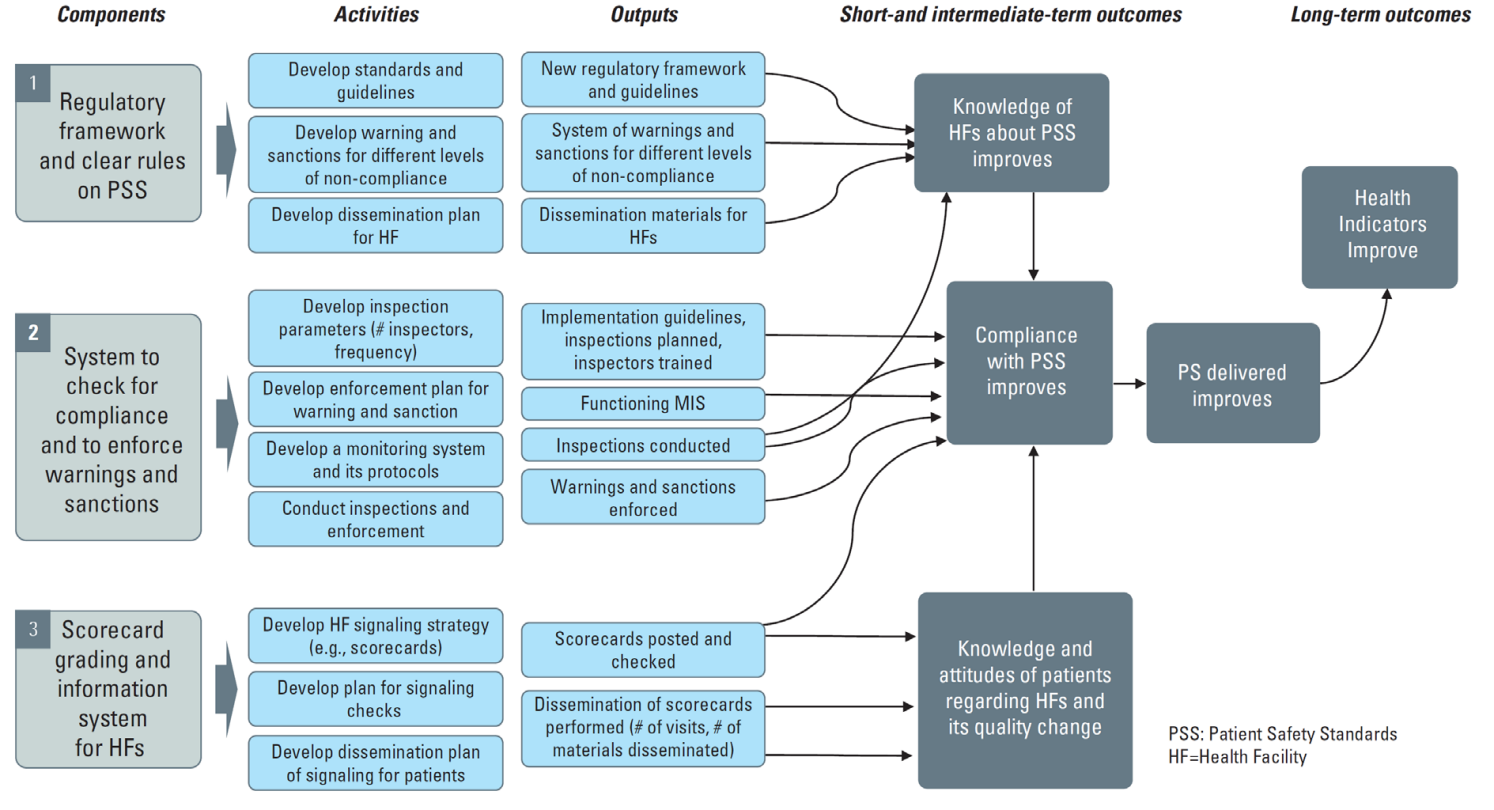
Theory of Change for Joint Health Inspection Pilot. Abbreviation: PS, patient safety. Source: KePSIE Evaluation Team.

 The KePSIE team outlined the activities, outputs and hypothesized causal pathways in a Theory of Change (Figure 1) for the JHI pilot. The intervention had three broad components: (*i*) a regulatory framework with clear guidelines on minimum patient safety standards and sanctions; (*ii*) a system for tracking compliance through inspection and enforcing warnings and sanctions, with an accompanying online monitoring system; and (*iii*) (in arm 2 only) performance scorecards indicating compliance category posted on facility walls with leaflets explaining these provided for patients. These components were expected to improve the knowledge of health facility staff and patients, and increase staff incentives for compliance, ultimately improving patient safety and health outcomes. Inspections were conducted using an electronic Joint Health Inspection Checklist (JHIC) on a tablet, which automatically generated a facility score. Inspectors gave the facility a brief summary report indicating the scores, with a full physical report provided at a later date. During the one-year pilot, 2138 inspection visits were made to intervention health facilities, out of which 1670 resulted in successful inspections. A total of 385 closure visits were made resulting in 176 facilities being closed physically, together with many departments within other facilities.

 The RCT’s focus was on the impact of the intervention on JHI scores. Preliminary results showed that inspection scores for facilities in the intervention arms were 15% higher compared to normal practice (41% vs. 35% compliance), with larger improvements in private facilities (19% increase vs. 7% in public).^19^ There was no difference between intervention facilities with and without scorecards.

 There is increased recognition that understanding the change processes behind such results, and how interventions actually work (or fail to work) is extremely important.^20-23^ In doing this, it is essential to listen to the voices of the actors directly involved, to understand their views, perceptions and experiences. In this paper we present the findings of a qualitative evaluation of JHI pilot implementation, conducted independently from the KePSIE RCT. We draw on interviews with those involved in the pilot, including staff from the MoH and World Bank, regulatory agencies, county governments and facilities. We focus here on the inspections and closures (public display of scorecards and community perceptions will be covered in a separate paper). The objective is to assess how the JHI pilot was implemented in practice, identifying key facilitating or hindering factors, in order to draw lessons for regulatory policy and practice.

## Methods

###  Study Setting

 The three pilot counties were selected because they contained a wide diversity of market sizes and to ensure a representative mix of regions.^17^ Key county characteristics are summarized in Table 2. Data collection for this qualitative study was conducted in these three counties and in the capital city, Nairobi.

**Table 2:** Characteristics of Study Counties

	**Kakamega**	**Kilifi**	**Meru**	**National **
Region	Western	Coast (South)	Central/Eastern	Not applicable
Size (square km)	3020	12 540	7006	580 876
Population	1 867 579	1 453 787	1 545 714	47 564 296
Population density (per square km)	618	116	221	82
Poverty levels (% below national poverty line)	49.2%	58.4%	31.0%	45.2%
Number of health facilities	316	285	513	>9000

Source: County size and population data,^24^ Poverty levels,^25^ Number of health facilities.^26^

###  Study Design and Data Collection

 The study employed a qualitative design. We began with a workshop to review the JHI Theory of Change (Figure 1) in February 2018, attended by MoH officials, World Bank staff, regulatory agency staff, county officials and inspectors. They highlighted other key factors influencing implementation, and informed the development of in-depth interview topic guides. The guide covered respondents’ views and experiences of each aspect of the inspection reforms and their perceptions of impact, with tools adapted slightly for each interviewee type to reflect the nature of their involvement (tools are provided in Supplementary files 1-4). Interviewees were contacted by phone to schedule appointments. We conducted 77 in-depth interviews with purposively selected stakeholders at the national, county and facility levels who were directly involved in the implementation of the inspection reforms. At the national level, we interviewed MoH officials, regulatory managers from the regulatory agencies and a representative of the private sector umbrella body; at the county level, county officials, inspectors, and World Bank field staff; and at the facility level, staff or managers present at the time the inspections were conducted (Table 3). Of the 77 interviewees, 33 were female (25/51 facility staff, 2/6 county managers, 0/5 inspectors, 3/5 World Bank field staff, 3/10 national level interviewees).

**Table 3:** Characteristics of Interviewees

**Interviewees **	**Kakamega**	**Kilifi**	**Meru**	**National**	**Total**
**County level**					
Health facility staff	17	17	17	-	51
County MoH managers	2	1	3	-	6
Inspectors	1	2	2	-	5
World Bank field staff	1	2	2	-	5
**National level**					
MoH officials	-	-	-	2	2
Regulators	-	-	-	6	6
World Bank staff	-	-	-	1	1
Private sector representative	-	-	-	1	1
**Total**	**21**	**22**	**24**	**10**	**77**

Abbreviation: MoH, Ministry of health.

 Facility interviewees were selected to achieve a mix of facility ownership type (23 public, 20 private for-profit, 8 FBO), facility level (32 dispensary, 12 health centre, 7 hospital), and inspection scores.

 Following a 4-day training workshop, and piloting in one of the trial counties, two authors with considerable qualitative research experience (ET and IK) conducted the interviews between May 2018 and April 2019 until data saturation was achieved. All interviews were conducted in English and took an average of 45 minutes to one hour. Interviews were audio-recorded where possible, and notes taken. No interviewees refused to participate, but we were unable to secure an appointment with one national actor and 10 health facilities; the latter were replaced with similar facilities in terms of ownership, level and inspection scores.

###  Data Management and Analysis

 Interview data were transcribed in English and exported into NVivo 12 for content management. Analysis was done using the framework approach.^27^ The process began with the research team reading the transcripts and interview notes to familiarize themselves with the data. An initial thematic framework was developed in a team workshop, drawing on the study objectives and the insights from our workshop on the JHI theory of change, and from themes emerging from the data. Co-coding of selected transcripts by CG, ET, IK and FW was used to refine the coding framework, with all transcripts then coded by either EK, IK or DO (coding tree provided in Supplementary file 5). Codes were organized into thematic groups to identify key facilitating and hindering factors for effective inspection. Preliminary findings were discussed with national and county level participants during a national workshop held in September 2019.

## Results

 We present findings on factors facilitating or hindering the implementation of the JHI pilot at the: (*i*) Micro level – concerning the facility-level experience of inspections; (*ii*) Meso level – concerning the operation of the inspection system; and (*iii*) Macro level – concerning links with the broader health system.

###  Micro-level

 Three key themes were identified at the micro level, related to the perceived fairness and objectivity of inspections, the inspection culture, and the potential for bribery.

####  Fairness and Objectivity

 An important facilitating factor was that nearly all respondents felt the JHIs were generally fair. National actors observed that fairness and objectivity were emphasized in the design and implementation of the JHIs, and in training of inspectors. Facility staff appreciated receiving a copy of the JHIC prior to inspections, and a summary report at the end outlining areas for improvement, which were perceived to have enhanced transparency, improved awareness of standards, and increased confidence in the process.

*“The checklist is now more objective, yeah, it doesn’t depend on the operator who is the inspector, yeah, so there is no room for subjectivity there, it’s an objective checklist and it’s a matter of you tick, if it’s a yes or no answer, do you have it or you don’t have it*…*”* (National actor 4/1).

*“But these ones have a checklist and I have a copy…even if I don’t read it today, I can go back to the checklist, look at it and see what I can do” *(Private dispensary staff 1/4).

 Respondents across all categories liked that the checklist was electronic and could compute scores automatically, which they felt standardized inspections and reduced bias. Facility staff were generally happy that inspections were carried out in their presence. This made the scores more acceptable.

*“... yeah it’s like they had trust in the electronic bit of it, yeah you could see also the excitement when you are giving them to sign eeeh ! Then at the end then the results they were like ‘ Aaah aah, the results are there, so I got 75% so I got a 60%’ so you can see maybe they appreciate that one”* (Inspector 3/2).

*“The scoring was like a team work. Even the people [facility staff] were involved. If you don’t have this, you know… that’s why the score is low. So, there was, there were very few incidences where they feel like they were unfairly treated” *(County manager 1/2).

 Facility staff and county managers reported no interference with inspections by County Health Management Teams. A JHI appeals process was set up, which some inspectors and World Bank field staff felt was another way of promoting fairness, allowing for re-inspection by a different inspector. However, in practice no appeals were made about disputed scores, although several facilities called the MoH coordinator to contest closures.

 Facility staff had varied opinions on the inspection scores they received. Some felt they were fair and reflective of their performance, while others felt they were harsh, particularly the first inspection. Many staff felt the checklist punished smaller facilities, and proposed additional customization. This partly reflected limited understanding of the skip process in the checklist whereby smaller facilities were not penalized for lacking certain services such as a labour ward, but facility staff did not always realize this. However, the concerns were also directed at items required for all facilities which they deemed irrelevant, for instance, expecting a small facility with few staff to have an organogram.

*“Maybe what they can do if you can design a checklist specifically for a dispensary, a checklist specifically for a hospital so that it is very particular*” (Public dispensary staff 2/16).

 Some small private for-profit facilities said they did not have the capacity to meet some JHIC standards, asking that longer grace periods be allowed to enable them to raise funds.

*“The private facilities they would cry much and say that whatever the checklist is asking for from my small facility, they are asking me for a vacuum extractor, they are asking me for things like what, some equipment, it is so expensive for me because I am the person who is supposed to foot that bill” *(World Bank field staff 1/2).

 Another concern raised by various actors was that poor scores were an unfair reflection of performance of staff working in public and some faith-based facilities, who had little control over finances and therefore lacked capacity to implement major changes (see macro level factors below). Finally, one private for-profit facility manager worried that having a single inspector conducting inspections (rather than an inspection team) could result in bias or favoritism, but this point was not raised by other actors.

####  Supportive Culture of Inspections

 All respondents observed that inspections under the previous system were intimidating, harsh and focused on fault finding. Facility staff said these visits, often conducted by multiple inspectors from different regulatory agencies, felt like “police raids,” at times leading to arrests. They observed that the JHI took a more supportive approach, something which other actors noted was emphasized during inspector training.

*“ So from the onset there was going to be a paradigm shift in terms of how the inspectors viewed the inspection, then how the service providers would actually be included, so that they would look at these as a form of a continuous process of improvement rather than an act of enforcement” *(National Actor 4/2).

 “*Most of them [facility staff] were welcoming, most of them were positive. You can remember that our approach was different from the regular inspections so the approach itself gave them an opportunity to welcome us and listen to us, so we made quite a few friends in there during the inspection*”(Inspector 2/1).

 Nearly all facility staff confirmed that the change in culture was felt on the ground, adding that inspections were now more supportive, friendly and designed to improve compliance. They appreciated that inspectors took time to explain the process and respond to queries.

*“These inspections have been friendly. Second, they teach us a lot, they make us conscious. Some things that we have forgotten these inspections make us very perfect because I know when they come they are going to ask me these questions so where I was failing I try to do some corrections there. I like them” *(Private dispensary staff 2/2).

 Facility staff also appreciated being allowed to interrupt inspections to attend to patients where necessary. The friendly nature of the relationship with inspectors was illustrated by facilities calling for re-inspections after implementing improvements. However, there were a few exceptions, where facility staff complained of joint health inspectors being harsh and combative.

####  Reduced Potential for Bribery

 All respondents acknowledged that inspector bribery was a common feature of the previous inspection system. This was attributed to the intimidating nature of inspections and the absence of mechanisms for holding inspectors accountable. In contrast, most respondents said they had not experienced cases of bribery with the JHIs, which they attributed to their friendly, standardized and transparent nature. Other measures linked to reduced bribery included random quality checks by World Bank field staff to verify inspection scores, the rule that prevented an inspector from conducting two consecutive inspections at a facility, the visibility of scores data on the electronic monitoring system, and the emphasis on reputation for integrity during inspector recruitment.

*“The inspections which have been done in the past, most of them were based on money and you will not get advice from them. Once they come they’ll maybe want the inspection fee and whatever. You get the receipt for the inspection fee and after they ask for bribes so you give them so they can leave and you continue with your work. So what I can say these last two [JHI] inspections have been perfect because they came purposefully to advice people” *(Private hospital staff 1/7).

 However, there were some reports of inspectors trying to solicit bribes and some inspectors reported being offered bribes to change inspection scores or leave a non-compliant facility open. One facility said they suspected their pharmacy was closed because they did not bribe.

###  Meso-level 

 Two major hindering/facilitating themes were identified at the meso-level – the management and logistics of the inspection system, and the process for closing departments or whole facilities.

####  Operational Management and Logistics

 At the inspection system level, several operational factors were linked to successful implementation of the inspections. First, inspector training was said to have been rigorous and thorough, involving intensive classroom exercises and practical experience.

*“Okay, the training was intensive, and it was good and well-articulated, and it was detailed. So that when we left there, we were able to carry out the inspections without any hiccup or anything” *(Inspector 3/1).

 Secondly, inspectors noted that the electronic format of the JHI made inspection processes easier, allowed instant generation and issuance of summary reports to the facilities, and enabled inspectors to easily upload inspection reports to a central server.

*“Okay let me tell you in general I think that’s the best thing ever, I think that’s the best way to go is using the electronic bit of it because one it made my work very easy. I just go there, I key in my findings then at the end I get my results” *(Inspector 3/2).

 World Bank field staff noted that their post-inspection checks helped strengthen implementation. These included random spot checks to confirm that facilities in arm 2 were still displaying inspection scorecards, and that closed facilities had not reopened, and quality checks to countercheck JHIC scores in random facilities.

 However, some logistical challenges were noted. For all counties, inspection delays resulted from bad weather and terrain, vehicle breakdowns, and delays in releasing funds for fuel and other operations. While two counties had MoH vehicles, the third was provided with a county vehicle, which was frequently recalled for other county functions.

 Secondly, some inspectors felt they should have been more involved in making daily inspection visit plans. Route planning was done by World Bank field staff, with urban facilities inspected first, as they were located closer to each other facilitating monitoring in the early stages. Some inspectors felt inspections should have started in more remote facilities during the initial dry months of the year (January and February), with more accessible urban facilities inspected during the rainy season. In addition, inspectors mainly shared one vehicle in each county, meaning they had to be dropped and picked at different facilities each day along a planned route. This proved challenging as sometimes inspectors had to wait for long periods to be collected after finishing their inspection, which was felt to be inefficient and sometimes posed a security threat for inspectors who had recommended facility closure.

*“You know there were instances where … if I’m the first person I reach my facility the next person will reach his facility after one hour, you get that? That is before the third person is dropped, so … even before the third person is dropped, I am through with the inspection and then I start waiting, I wait for three to four hours and that was very frustrating” *(Inspector 2/1).

####  Closure and Grace Periods

 According to the JHI protocol, facilities without a valid license were to be closed immediately. The process entailed inspectors passing a closure notice to the county government, which would then authorize their PHOs to execute the closure within two weeks and display a closure notice at the facility. A similar process was to be followed if specific departments, such as lab or pharmacy, lacked their own license. However, the closure process faced challenges. Early testing of the JHIC revealed that a large proportion of facilities lacked valid licenses and would have had to be closed, likely affecting overall service provision. For this reason, the KePSIE Task Force introduced 90-day grace periods for facilities with expired licenses, as long as they had qualified staff and expressed an intention to renew. In addition, closures were affected by nationwide strikes by public sector doctors (December 2016 to March 2017) and nurses (June to November 2017), which paralyzed public services. The fear was that closing private facilities would have negatively impacted access.

*“…when we wanted to close and in the dynamics in the ministry we had nurses strike then we had the healthcare workers strike and then we closed a facility that was faith-based. They started calling that we don’t have anywhere to go and here you are closing so once in a while we would get negative publicity…negative reaction from the counties” *(National actor 4/3).

 Even following grace periods, interviewees observed that county PHOs did not carry out closures as envisioned. As a result, the JHI protocol was changed and the role transferred to the MoH coordinator, who would travel from Nairobi periodically to close facilities.

*“So, for example the protocol was that initially when a facility was reported for closure by an inspector someone from the county must go and close it within the next 24 hours, you know 24 or 48 hours. Well the county didn’t do that, that was not even remotely feasible at all..... we realized that the only way that they were going to happen is if someone from the ministry, from the national government went…” *(National actor 4/4).

 The shift of this role to the MoH coordinator caused delays of up to six months in effecting closures, with some inspectors complaining that this reduced their legitimacy and morale. Several reasons were given for the failure of county staff to implement closures. In one county in particular, there were five changes in senior county health management staff during the year, requiring repeated orientation about JHI. Secondly, public officials felt it was inappropriate to close public facilities, even where their performance was extremely poor. However, they also failed to implement private facility closures. Some PHOs were said to be reluctant to take up this new role which they felt was not part of their job description, also complaining of lack of funds for transport.

*“…it took a very long time for this [PHO] to follow up, because every time we would go back it was like reminding him we had given you this and this and this, did you follow up or anything and he was like most of the time – ‘am not paid to do this work, I’m taking time out of my normal duties,’ so I don’t know whose duty it is because he is the public health officer for the county or the sub-county” *(World Bank Field Staff 3/2).

 Some respondents felt these complaints reflected deeper concerns and conflicts of interest on the part of county staff, who were part of the same community as the facilities they were required to close. This could lead to concerns for their safety following closures; and reluctance to close facilities owned by county officials.

*“…we had challenges because some of the officers from the county they say this is a facility in my village if I accompany you to close it, you guys will go back to Nairobi, you will leave me here in the village, this person will come for me in the village, so there was that challenge in terms of the relationships the county officers had with those facilities. Some of the county officers own those facilities, so there was a bit of conflict of interest in terms of that and that’s why our recommendation was the national regulatory body…be the one to go and enforce the closure” *(National actor 4/1).

 Some interviewees reported concerns over the credibility of PHOs, who had a reputation for collecting bribes from facilities operating unlawfully.

*“And also in some instances you would tell the PHO that this facility needs to be actually closed and maybe he had some relationship with that facility because when we went to the facility the providers told us that the PHO takes money from them on a monthly basis” *(World Bank Field Staff 3/2).

 Broader political influence and interference were also reported to have affected enforcement of closures. One facility staff reported that closing a public facility in an area perceived to be an opposition stronghold could be interpreted as an attempt to portray local leaders in bad light.

*“People may interpret it like if you’re in opposition zone people in the national government is trying to suppress us especially now a politician goes and repeat that in a podium it will be counterproductive to the national government” *(Public hospital staff 2/15)

 There were also a few cases where facilities recommended for closure continued operating because the owner had political influence.

*“And there were others who really, they didn’t care because there was another facility somewhere in [large town] had a closure scorecard on the door but it used to operate normally, yes they didn’t care about the closure I think he knew some big people in the county offices so he wasn’t afraid of the scorecard” *(Inspector 3/1).

###  Macro-level 

 The operation of the inspection system was interconnected with the broader health system in ways that both facilitated and hampered implementation, with links to licensing, facility resources and the buy-in of the regulatory agencies.

####  Links to the Licensing System

 The JHIC required that facilities have a general license, department licenses (eg, laboratory), and licenses for professional staff. Facility and department licenses were renewed annually and professional licenses every two to three years. Licensing and inspection processes were independent of each other, with the former carried out by the respective regulatory agencies.

 The majority of facility staff raised concerns over the process and cost of licensing, and the consequences for inspection. Satisfaction with licensing processes varied substantially across regulators, with some being more efficient than others. For instance, while some regulators had online renewal processes, others required staff to travel to Nairobi to renew licenses.

*“It’s cumbersome, really cumbersome because you see you have got to go all the way to Nairobi…for the license which it can be done maybe online or maybe decentralized or devolved down to the county level” *(Private dispensary staff 3/14).

 There were complaints that some regulators delayed issuing licenses after payment, sometimes for over a year. There were also complaints over multiplicity of licenses, with arguments that these should be streamlined into a single license.

*“But then now, there are several other multiple players each one wanting a cost and the cost of opening a facility, because now for you to have an x-ray you have to need some license from Radiation Protection Board, and the laboratory will require licensing from Kenya Medical Laboratory and Technologists Board, the nurses will of course get licensing from the Nursing Council, clinical officers from Clinical Officers Council. So, it brings, this facility now gets overtaxed and I think to me, it is multiple taxation” *(Public hospital staff 2/15).

 To complicate matters further, public facilities were registered directly by the MoH, independently of regulatory bodies and county authorities, and the system for licensing departments in public facilities was yet to be finalized.

####  Access to Resources for Public Facilities

 Public facility staff expressed great frustration at receiving low JHI scores, which they felt reflected badly on their performance, while they had little control over resources and could not undertake important improvements, such as increasing staff, and purchasing equipment.

*“Me as the in-charge for example, I rely on the goodwill of the County and despite of how much we advocate, I can only move as far as they can support me and if they don’t support me then I might remain grounded however much my good intentions might be. I might say I want this, and if they don’t finance me to do that, if they do not give me more health workers for example in a certain department, then there is nothing much I can do” *(Public hospital staff 2/15).

 It was evident from several interviewees that this really demotivated some public facility staff.

####  Buy-in From Regulatory Bodies

 A final macro-level factor key to the success of the inspections was the buy-in from regulatory bodies. Prior to JHIs, the regulators had been operating in silos, carrying out fragmented inspections. Although some agencies had been cautious or even hostile to a joint approach when it was first proposed, they now felt the JHIs had enhanced cohesion and harmonized inspections.

*“For me I always looked at, number one, the joint health inspection checklist helped reduce clutter in terms of harmonizing all boards to be able to design a tool that hits all areas, I felt it made the process more efficient. I feel a lot of hospitals are cheated when one inspector comes, the other one, inspector comes, the other one inspector … it’s time consuming and so on”* (National actor 4/5).

 The buy-in was linked to the inclusive JHIC development process which involved all agencies, and the reassurance that their autonomy as individual agencies had not been removed.

*“Well, I would not really say that it has affected autonomy, it has only impacted on inclusion because the Acts were not amended and therefore it means that the powers that are still within those Acts are still there and it did not stop for example a member of a regulatory board or council again walking into that particular county if they deemed fit” *(National actor 4/2).

 Most regulatory bodies also appreciated increased license applications and renewals as a result of the inspections, which increased revenue for their agencies.

## Discussion

 The JHI pilot was praised by Kenyan stakeholders for streamlining fragmented inspections and harmonizing the activities of the regulatory agencies. The pilot was of insufficient duration to assess the full impact of the risk-based and responsive regulatory elements of the JHI, as less than two thirds of facilities received a second routine inspection visit during the one year of implementation. However, the KePSIE RCT demonstrated a significant increase in JHIC compliance during this period, despite a challenging context including sustained health worker strikes and two general elections. JHIs are now being adapted for national scale up in Kenya, while the innovations have generated interest from other African countries, with several making study visits to Kenya to learn more.

 We set out to study lessons for future policy and practice from this pilot by identifying key factors that facilitated or hindered its implementation. Before discussing our findings, we note a number of potential limitations of this research. First there was a risk of social desirability bias when asking respondents about the performance of JHI. Actors may have wanted to present themselves in a good light, for example, facilities presenting themselves as eager to comply with regulation, or inspectors and county actors presenting themselves as having fulfilled their roles and operated with integrity. Moreover, while our fieldwork team were independent of the inspection reforms and the KePSIE RCT, investigators FW and NG played substantial roles in JHI implementation. While having such “insiders” in our study team was a strength in terms of their tacit knowledge and access to interviewees, it is possible that this led some interviewees to feel that favorable responses on JHI would be well-received. We aimed to address this by stressing that no information linkable to individuals would be presented, by offering the option of not being audio-recorded, and by reflecting on our positionality as a team, and note that many interviewees did offer critical perspectives. Secondly, the JHI pilot was undertaken in 3 counties only, and as part of an RCT. The approach might work differently if implemented in different counties, or on a larger scale where close oversight was not possible. In particular we believe the intensity of health facility inspection to be unprecedented in this type of health system, and its replicability without external support is unclear. On the other hand, larger scale, long-term implementation could improve effectiveness as the regulatory reforms become institutionalized and better understood. Finally, we have focused here on factors affecting the implementation of the inspections and have not explored the implications for patient safety and quality of care. There is often poor correlation between the structural quality measures included in inspection checklists and clinical quality of care ^28^, indicating that an effective inspection system alone may be insufficient to improve patient outcomes.

 A starting point for our analysis was the JHI Theory of Change (Figure 1) which set out the technical elements of the JHI. Together with the financial, human and physical resources required, these technical elements could be termed the “hardware” of the reforms, including the regulatory framework and guidelines, protocol for warnings and sanctions, inspector training, monitoring system, and scorecards.^29^ What is striking from our findings is that an effective inspection system involves much more than these “hardware” elements; it depends on a wide range of relational and cultural factors – the “software” of the system^30,31^ – which have a crucial impact on the system’s perceived legitimacy, feasibility of implementation and enforceability. To allow others to replicate or learn from the JHI experience it is important to make these “software” elements explicit, and explore their roles.

 Starting at the micro level, the JHI Theory of Change emphasized the mechanism of improving the knowledge of health facility staff in order to improve their compliance. Our findings indicate that facility staff’s perceptions of the objectivity, fairness and transparency of the inspection system are central in influencing its legitimacy, and therefore another key mediating factor. Interviewees reported receiving the JHIC prior to inspection, conducting inspections in the presence of facility staff, and use of an electronic checklist as factors enhancing perceived legitimacy and thus support of the new system. These issues have been highlighted more generally in the regulatory literature from LMIC and high-income countries, with greater legitimacy of regulatory systems argued to lead to higher levels of compliance without very heavy investment in policing performance.^32-34^ For example, a systemic review of inspections, primarily in high income settings, underscored the importance of predictable and transparent inspection processes that are perceived as valid and reliable in encouraging change in response to inspection findings.^33^

 These design features were also potentially important in restricting the potential for bribery, together with random quality checks by World Bank field staff, and requiring different inspectors to conduct consecutive inspections. According to study respondents, bribery was rampant in the pre-JHI inspection system, echoing findings from other LMIC contexts.^14,35^ This had largely been curbed under the JHI, with the exception of a few possible cases. However, the generalizability of these results beyond the pilot should be carefully considered. World Bank field staff provided close oversight in the three counties, through both their support to JHI implementation and the RCT, which involved additional interaction with facilities for survey data collection. Given how commonly corruption is reported within Kenya’s public sector,^36^ it is likely that the inspection system’s ability to control bribery would be severely tested in a context of national scale up, without similar external oversight.

 Another key software element identified for JHI acceptance was the culture of inspections and the relationships that inspectors formed with facility staff. JHIs were viewed by facility staff as helping them improve as opposed to punishing them for non-compliance, and were said to be supportive and friendly. This approach was deliberately fostered by the JHI team and emphasized during inspector training. This resonates with recent work highlighting the “social dimension” of regulation and importance of relationships between regulator and healthcare staff in fostering change, while recognizing that there is a difficult line to tread between developing good relationships and maintaining sufficient critical distance.^33,34^ Emphasis on culture and relationships fits with a responsive regulation approach, and the compliance rather than deterrence approach to enforcement, where regulators begin by drawing on education, advice, persuasion and negotiation,^7^ contrasting strongly with the punitive approach typical in many LMICs.^35^ Some facility staff still found the first inspection “harsh,” emphasizing the potential role for a purely advisory first visit.

 The results also highlighted the critical nature of practical considerations in effective implementation of an inspection system. Implementing regular inspections and enforcing closures in numerous widely dispersed facilities is a substantial logistical challenge. It is essential to consider carefully the need for systematic planning, efficient route scheduling, transport availability, human resource management, and quality assurance, all of which need to be resourced through a realistic budget, with contingency for unexpected costs such as vehicle breakdown. Even during the relatively well-resourced JHI pilot, vehicle breakdowns, unfavourable weather, poor roads and inefficient scheduling were said to have substantially slowed down inspections.

 Our findings show how dependent the effectiveness and legitimacy of an inspection system is on other institutions within the broader health system. Regulation is effectively a shared function between the national MoH, the county health teams, and regulatory bodies, which all had important supportive and enforcement roles to play. However, gaps were reported in some aspects of these roles, including enforcing closures, licensing facilities and departments, and financing public facilities. Starting with closures, even if emphasis is placed on support and encouragement of facilities, at some point sanctions are required to increase incentives for compliance and protect patients from the poorest quality care. The closure of non-compliant facilities was a key component of the JHIs but initial plans for County Governments to undertake this were not executed, and KePSIE data indicated that a high proportion of closed facilities reopened without permission.^19^ While this may partially have reflected lack of resources and concerns for public access to services, it appears that it also reflected personal and political conflicts of interest for county officials, reflecting a tension between local ownership and independence in regulatory implementation. As a result, the closure role was transferred to the MoH Coordinator, who personally visited each facility requiring closure. This caused substantial delays, and it is unclear how effectively this could be replicated at a national scale.

 Possession of an up-to-date license for facilities and departments was a fundamental point of JHI compliance, but the licensing systems were managed separately by the individual regulatory agencies. The inspection reforms clearly stimulated greater demand for license renewal, and therefore licensing revenues. However, for some (though not all) regulators the bureaucracy and inefficiency associated with licensing processes led to substantial delays in renewal, which were seen as unfair by facilities where they led to warnings and sanctions. This emphasizes the importance of streamlining licensing procedures, by use of online application processes and/or delegation to regional offices. There were also calls for a single license to be introduced for health facilities, incorporating all departments. This may face resistance from some regulators if they feel their autonomy or revenues are threatened. However, Kenya is considering moving in that direction under the guidance of the recently established Kenya Health Professionals Oversight Authority.

 This study raises important questions about the regulation of public sector facilities. Previous inspection systems have focused on the private sector. The inclusion of public facilities in the JHI was seen as important in increasing fairness by ensuring that private facilities did not feel unreasonably targeted. However, in many cases the capacity of public facilities to improve compliance was limited by their lack of control over resources. Health centers and dispensaries in particular had no facility-level budgets, nor could they retain user fees for their own use. They reported feeling demoralized by poor scores in repeated inspections when they had been unable to make changes. It could be argued that the County Governments themselves would have been more appropriate recipients of the inspection reports, and/or that lower level public facilities should be provided with devolved budgets. Inspection of public facilities also raised concerns about conflict of interest, and how effectively the government could be expected to regulate itself.

**Figure 2:**
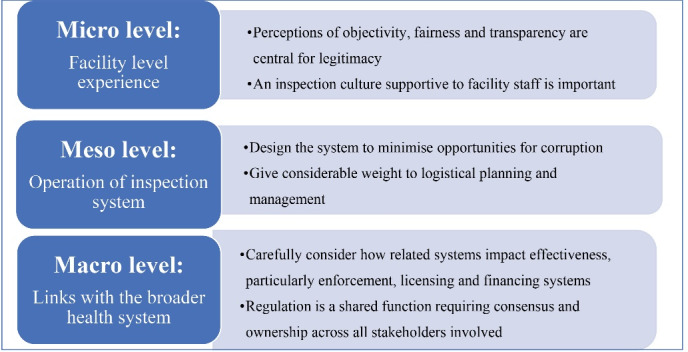
Facilitators of Effective Health Facility Inspection.

 In sum, there is a great deal that can be learnt from the JHI experience in Kenya, of relevance not only to health facility inspection, but also to healthcare regulation more broadly (see summary in Figure 2). The JHI contained substantial “hardware” innovations, in terms of the harmonization of standards across regulatory agencies, the risk-based and responsive design of the inspection protocol, the electronic checklist, the online monitoring system, and various design elements to minimize corruption. We have highlighted here the additional “software” elements which were both key to its success and could hinder its performance, particularly perceptions of fairness and legitimacy, a supportive inspection culture, strong planning and management, and high-level buy-in. Finally, it is essential that inspection systems are not considered in isolation, as their effectiveness is heavily influenced by the related systems for enforcement, licensing and financing. We recommend that those looking to reform or improve enforcement of health facility regulation carefully consider these cultural, relational and institutional dimensions.

## Acknowledgements

 The authors would like to acknowledge the support of Gilbert Kokwaro of Strathmore University, Guadalupe Bedoya and Amy Dolinger of the World Bank Group, the Kenyan Ministry of Health (Directorate of Health Standards, Quality Assurance and Regulation), and all study participants for providing valuable insight and information.

## Ethical issues

 This study was approved by Strathmore University’s Institutional Review Board (0194/18) and the Ethics Committee of the London School of Hygiene and Tropical Medicine (14647). Informed consent was sought from participants. Verbal consent was sought as similar studies had found that participants worried that written consent could be incriminating when discussing legal and regulatory issues.

## Competing interests

 Authors declare that they have no competing interests.

## Authors’ contributions

 CG, FW, and NG conceptualised the study, ET and IK collected data and performed preliminary analysis. All authors were involved in the final analyses. ET developed the first draft of the manuscript. All authors contributed to subsequent and final drafts.

## Funding

 The study was funded by the Health Systems Research Initiative (Medical Research Council, Economic and Social Research Council, Department for International Development, Global Challenges Research Fund, and Wellcome Trust), grant #MR/P014291/1. The funders had no role in study design, data analysis, decision to publish, drafting or submission of the manuscript. The views expressed in the paper are of the authors and not of the organizations they represent.

## Supplementary files


Supplementary file 1. Interview Guide – Facility.

Supplementary file 2. Interview Guide – Inspectors and World Bank Coordinators.

Supplementary file 3. Interview Guide – National Actors.

Supplementary file 4. Interview Guide – World Bank Staff.

Supplementary file 5. Coding Tree.
