# Supplementary material for: What Lies Behind Successful Regulation? A Qualitative Evaluation of Pilot Implementation of Kenya’s Health Facility Inspection Reforms
Source: Int J Health Policy Manag. 2021 Aug 25;11(9):1852–62. doi: 10.34172/ijhpm.2021.90 (PMC9808232; doi:10.34172/ijhpm.2021.90)
Supplement: Supplementary file 1 — Interview Guide – Facility. [file ijhpm-11-1852-s001.pdf]

Article title: What Lies Behind Successful Regulation? A Qualitative Evaluation of Pilot Implementation of Kenya's Health Facility Inspection Reforms

Journal name: International Journal of Health Policy and Management (IJHPM)

Authors' information: Eric Tama[1]*, Irene Khayoni[1], Catherine Goodman[2], Dosila Ogira[1], Timothy Chege[1], Njeri Gitau[3], Francis Wafula[1]

[1]Institute of Healthcare Management, Strathmore University Business School, Strathmore University, Nairobi, Kenya.

[2]Department of Global Health and Development, London School of Hygiene and Tropical Medicine, University of London, London, UK.

[3]World Bank Group, Nairobi, Kenya.

(*Corresponding authors: etama@strathmore.edu)

Supplementary file 1. Interview Guide - Facility

Facility KePSIE inspection details (complete before visit):

**Dates of inspections and scores given**	
**Any closures and dates**	
**In scorecard arm? (yes/no)**	
**Scorecard currently displayed?**	

Respondent Profile

**Name**	
**Gender**	
**Age**	
**Qualification**	
**Name of Facility, Level, Sector**	

Respondent profile

1. What is your current position (Probe - what does it involve, management role)?
2. How long have you been in this role? What other roles have you held in this or other health facilities?

Experience of regulation

3. In 2017, a new type of facility inspections was introduced that uses the JHICs.
    a. Are you aware of the JHIC? Do you have a copy? Have you read through it?
    b. We understand your facility had (..) inspections during 2017 – were you present or aware of these?
4. What are your views on the JHIC and the 2017 inspections?
    a. Do you see any difference(s) between the 2017 and older inspection systems? What type?
    b. What are your views on the content of the inspections? (do you feel it is relevant to your facility level?)

c. Are you aware that there are different scores for different questions? What are your views on this?
d. Are you aware that the score a facility gets determines the duration before the next inspection? What are your views on the time given until the next inspection?
e. What are your views on the time the inspection takes at the facility? Is it disruptive to you?
f. What do you think of the inspectors and how they carry out the inspections?
g. What did you think of the inspection reports? Did you receive them after every inspection, understand them, find them useful?

5. We see your facility was given a scores of (…) in (month) and (…) in month……..;
    a. What do you think of those scores? Were they fair?
6. What are your views on the licensing process for the health facility?
    a. What are your views on the licensing process for departments within the facility? (Ask about the lab, pharmacy)
    b. What are your views on the licensing process for professionals/staff?
7. (Where relevant) We understand that you had your facility/department closed:
    a. How long did the facility/department stay closed in practice?
    b. How did you feel about the closure?
    c. Did you lose revenue? How much?
8. Do you think facilities/departments without valid licenses should be closed? Why?
9. (If in scorecard arm) What do you think about the display of scorecards at the facility?
    a. What do you think of the scorecard design and content?
    b. What do you think of the location where the scorecard is displayed?
    c. Have you had patients approach you to discuss the scorecard? What issues did they raise? Do you think the scorecard influences patient choice of facility?
    d. Did having a scorecard displayed influence your actions as a facility?
    e. What do you think should be done differently in relation to scorecards?

Impact of reforms

10. Has anything changed at this facility as a result of the inspections? (Probe what has changed,)
11. Have the inspection requirements led to additional costs to this facility? What type? How much?
    a. To change practices to comply with regulation – e.g. equipment, infrastructure, staffing, overheads
    b. License fees, record keeping

    c. Penalties for non-compliance, or loss of income.
    d. In each case were these costs incurred purely to meet regulatory requirements?

12. Which issues highlighted in the last inspection have you not changed and why? How much would it cost you to have them changed?
13. Do you think the changes you have made have had an impact on patient safety, staff safety and quality of care? How?
14. Overall, what are your thoughts on the value of the joint health inspections when compared with the previous ways of doing inspections? (Probe – value in patient safety, quality of care, performance of legitimate businesses etc)
15. Would you say there have been negative impacts that resulted from the inspections? Which ones?
16. Sometimes inspectors like to ask for "kitu kidogo". Has this ever happened to you? Did it happen in the inspections carried out before 2017? What about in 2017?
17. Do you receive inspections from any other bodies (e.g. NHIF)? What is your view on these? How do they affect your practices? How do they compare to JHIC ones?

Way forward

18. These new inspections have only been carried out in 3 counties so far - what do you think about scaling up these new inspections to all 47 counties?
    a. What would be the challenges?
    b. What components of the project should be adopted and strengthened?
    c. What should be done differently and how?
19. There has been discussion of whether the scorecard design could be improved – here are some ideas. What are your views on these?
20. Do you think there are other better ways to communicate inspection results to the community?
21. Do you have anything else you would like add?

Many thanks for your time – Do you have any questions for us?
