# Supplementary material for: What Lies Behind Successful Regulation? A Qualitative Evaluation of Pilot Implementation of Kenya’s Health Facility Inspection Reforms
Source: Int J Health Policy Manag. 2021 Aug 25;11(9):1852–62. doi: 10.34172/ijhpm.2021.90 (PMC9808232; doi:10.34172/ijhpm.2021.90)
Supplement: Supplementary file 2 — Interview Guide – Inspectors and World Bank Coordinators. [file ijhpm-11-1852-s002.pdf]

Article title: What Lies Behind Successful Regulation? A Qualitative Evaluation of Pilot Implementation of Kenya's Health Facility Inspection Reforms

Journal name: International Journal of Health Policy and Management (IJHPM)

Authors' information: Eric Tama[1]*, Irene Khayoni[1], Catherine Goodman[2], Dosila Ogira[1], Timothy Chege[1], Njeri Gitau[3], Francis Wafula[1]

[1]Institute of Healthcare Management, Strathmore University Business School, Strathmore University, Nairobi, Kenya.

[2]Department of Global Health and Development, London School of Hygiene and Tropical Medicine, University of London, London, UK.

[3]World Bank Group, Nairobi, Kenya.

(*Corresponding authors: etama@strathmore.edu)

Supplementary file 2. Interview Guide – Inspectors and World Bank Coordinators

**Name**	
**Gender**	
**Age**	
**Qualification**	
**KePSIE Role**	
**KePSIE Location/county**	

Respondent Profile

1. What is your current position (Probe - what does it involve, linkage to health/quality/regulation)?
2. How long have you been in this role? Have you held any other roles related to healthcare quality /regulation?

Role in regulatory reforms

3. How were you involved with KePSIE? (Probe - role in implementation, how long they were involved in this role? How did you get into this role?)
4. What do you think of the training for KePSIE? How does it compare to other trainings you have received?

The JHIC

5. What do you see as the key activities/elements of the joint health inspections?
6. What do you think of the Joint Health Inspections Checklist (JHICs) - What has worked well and why?, and what has not worked well and why?
    a. What are your views on the content of the inspections? Is it relevant to all facility levels and types/ownership?
    b. What do you think of the scoring system? (different questions in the checklist having more weight than others). Do you think the scoring system is fair?
    c. What do you think of the time between inspections and it being tied to scores?
7. How do facility in-charges react/receive inspectors?
    a. Do you face any resistance? Do they have any complaints?
    b. Do you think the inspections are seen as fair by the facilities? Is there any way facilities can appeal their score?

Score card design

8. What are your perceptions about the use of scorecards in facilities?
9. What do you think about the suitability and appropriateness of this score card design and content? (Probe ease of comprehension and catchiness)
10. Where is the best location for displaying the scorecard? (Probe- Why)
11. Have you seen any difference between facilities with and without scorecards?
12. What do facilities think of the display of score cards?

Licensing and closures

13. What do you think of the licensing process for:
    a. The facility?
    b. The laboratory
    c. The pharmacy?
    d. Individual staff?
14. What do you think of the closure of:

a. Unlicensed facilities
b. Unlicensed departments
c. Should it be done?
d. What do you think of the process of closure?
e. How do facilities react to closures?

Deviation and Variation

15. Have there been deviations from the way you were trained and how inspections happened in the field? What deviations and why?
16. Have you seen any variation in the inspection process:
    a. Across facility types (public/FBO/private)?
    b. Across facility level (hospital/health centre/dispensary)?
    c. Across counties?
    d. Between inspectors?
    e. What would you say are the reasons for these variations?
    f. What would you say has been the effect of these variations on the overall goal of the inspections? (Probe both positive and negative effects)

Impact of reforms

17. Do you think the inspections have affected facility practices? (probe what practices have changed)
18. Do you think the inspections have had an effect at the facilities on:
    a. Patient safety?
    b. Quality of care?
    c. Staff safety?
19. What have you recommended for change and facilities have not changed? Why?
20. Would you say there were any negative impacts that resulted from the implementation of the inspections? Why?
21. How do you think the inspections are viewed by the counties? Do they have any complaints?

Employment conditions of inspectors

22. How was the relationship between the inspectors and World Bank coordinators?
23. What did you think of the terms and conditions of inspectors under KePSIE?
24. Do facility owners ever offer you tokens of appreciation?
25. We know there are occasionally cases of inspectors asking for kitu kidogo. Have you heard of such cases during KePSIE or the previous system of inspections?

Way forward

26. What are your perceptions about scaling up the project to all the counties?
    a. What would be the challenges?
    b. What components of the project should be adopted and strengthened?
    c. What should be done differently and how?
27. What do you think of the new system of inspections being implemented in 2018 as compared to the KePSIE inspections?
28. There has been discussion of whether the scorecard design could be improved – here are some ideas. What are your views on these?
29. Do you think there are other better ways to communicate inspection result to the community?
30. Is there anything else you would like to add?

Many thanks for your time – Do you have any questions for us?
