# Supplementary material for: What Lies Behind Successful Regulation? A Qualitative Evaluation of Pilot Implementation of Kenya’s Health Facility Inspection Reforms
Source: Int J Health Policy Manag. 2021 Aug 25;11(9):1852–62. doi: 10.34172/ijhpm.2021.90 (PMC9808232; doi:10.34172/ijhpm.2021.90)
Supplement: Supplementary file 3 — Interview Guide – National Actors. [file ijhpm-11-1852-s003.pdf]

Article title: What Lies Behind Successful Regulation? A Qualitative Evaluation of Pilot Implementation of Kenya's Health Facility Inspection Reforms

Journal name: International Journal of Health Policy and Management (IJHPM)

Authors' information: Eric Tama[1]*, Irene Khayoni[1], Catherine Goodman[2], Dosila Ogira[1], Timothy Chege[1], Njeri Gitau[3], Francis Wafula[1]

[1]Institute of Healthcare Management, Strathmore University Business School, Strathmore University, Nairobi, Kenya.

[2]Department of Global Health and Development, London School of Hygiene and Tropical Medicine, University of London, London, UK.

[3]World Bank Group, Nairobi, Kenya.

(*Corresponding authors: etama@strathmore.edu)

Supplementary file 3. Interview Guide – National Actors

**Name**	
**Gender**	
**Job Title**	
**Qualifications**	
**Name of Organisation**	

Respondent Profile

1. What is your current position? (Probe - what does it involve, linkage to health/quality/regulation)
2. How long have you been in this role? Have you held other roles related to healthcare quality /regulation?

Role in regulatory reforms

3. Have you/how have you been involved in the joint health inspections and the KePSIE project? (Probe - role details in design, roll out, implementation, evaluation and scale-up)
4. Who are the other key players in your institution involved in the joint health inspections / KePSIE project? (probe- what roles and how do they play them)

Policy design

5. What factors motivated the introduction of the joint health inspections?
6. How was the decision to introduce these reforms arrived at (Probe – who were the key actors/players in this decision, how were they involved etc?)
7. What do you see as the key activities/elements of the joint health inspections?
8. How were the joint health inspections designed to work in order to improve patient safety?

Implementation Process

9. What do you think of the Joint Health Inspections Checklist (JHIC) - What has worked well and what has not worked well and why?
    a. What do you think of having a Joint Health Inspection Checklist across all boards?
    b. What do you think about a solo inspector of any health cadre being recruited and trained to inspect a full facility?
    c. What are your views on the content of the inspections? Is it relevant to all facility levels?
    d. What do you think of the scoring system? (different questions in the checklist having more weight than others). Do you think the scoring system is fair?
    e. What do you think of the time between inspections and it being tied to scores?
10. What do you think of the training for inspectors?
11. What do you think of process of inspections in practice? (what worked well? What did not work well and why?)
12. What do you think about the online monitoring system? (Probe - do you ever use it? If so, how? Is it useful? What could be improved?)
13. What are your perceptions about the use of scorecards in facilities?
    a. What do you think about the idea of scoring and ranking facilities?
    b. What do you think about the suitability and appropriateness of the scorecard design and content? (Probe ease of comprehension and catchiness)
    c. Where is the best location for displaying the scorecard? (Probe- Why)
    d. Do you think scorecards affect facility performance?
    e. Do you think there are other better ways to communicate inspection results to the community?

Licensing and closures

14. What do you think of the licensing process for:
    a. The facility? Should this be applied to public facilities?

b. The laboratory?
c. The pharmacy?
d. Individual staff?
e. Was there a change in the number of facilities and personnel seeking licensing from the three pilot counties as a result of KePSIE?
f. Was there a change in revenue from licenses from the three counties?

15. What do you think of the closure of:
    a. Unlicensed facilities
    b. Unlicensed departments
    c. Should it be done?
    d. Do you think it is ok to leave a registered facility operating if it scored poorly (D or C)?
    e. What do you think of the process of closure?
    f. How did facilities react to closures?

Factors affecting implementation

16. Have you seen any variation in KePSIE implementation across facility types (public/FBO/private), facility level (hospital/health centre/dispensary) or across counties?
17. Have there been deviations from the way the regulatory reforms were designed to work? What type and why?
18. Do you think implementation would have been different if it was not being evaluated through an RCT? How?

Governance

19. What did you think about the role of counties in implementing the JHIs? (could their roles have been different? How much buy-in was there from counties?)
20. Do you think all boards and councils played an equal role in the process of developing the JHIs process? How about in the implementation of JHIs? (have the regulatory reforms affected your autonomy in any way?)

Impact of reforms

21. Do you think the inspections have affected facility practices? (probe what practices have changed, what has not changed, why?)
22. Do you think the reforms are seen as fair and legitimate by the facilities? By the counties?
23. Do you think there is difference in costs of regulation when compared to the previous ways of doing inspections? (if yes how? What type of costs?)

24. Do you think the reforms have led to additional costs to facilities? What type? (probe costs to change practices to comply with regulation, administrative costs, penalties, loss of income)
25. Would you say there were any unintended negative consequences/impacts that resulted from the implementation of the regulatory reforms? Why?
26. How do these regulatory reforms compare to the previous regulation strategy in terms of potential for bribing?
27. Overall, what are your thoughts on the value of the joint health inspection reforms when compared with the previous ways of doing inspections? (Probe – value in patient safety, quality of care, performance of legitimate businesses etc)

Way forward

28. What would you propose be done to improve the implementation of the project and achieve better outcomes?
29. What are your perceptions about scaling up the project to all the counties?
    a. What would be the challenges?
    b. What components of the project should be adopted and strengthened?
    c. What should be done differently and how?
30. Is there anything else you would like to add?

Many thanks for your time – Do you have any question(s) for us?
