# Supplementary material for: What Lies Behind Successful Regulation? A Qualitative Evaluation of Pilot Implementation of Kenya’s Health Facility Inspection Reforms
Source: Int J Health Policy Manag. 2021 Aug 25;11(9):1852–62. doi: 10.34172/ijhpm.2021.90 (PMC9808232; doi:10.34172/ijhpm.2021.90)
Supplement: Supplementary file 4 — Interview Guide – World Bank Staff. [file ijhpm-11-1852-s004.pdf]

Article title: What Lies Behind Successful Regulation? A Qualitative Evaluation of Pilot Implementation of Kenya's Health Facility Inspection Reforms

Journal name: International Journal of Health Policy and Management (IJHPM)

Authors' information: Eric Tama[1]*, Irene Khayoni[1], Catherine Goodman[2], Dosila Ogira[1], Timothy Chege[1], Njeri Gitau[3], Francis Wafula[1]

[1]Institute of Healthcare Management, Strathmore University Business School, Strathmore University, Nairobi, Kenya.

[2]Department of Global Health and Development, London School of Hygiene and Tropical Medicine, University of London, London, UK.

[3]World Bank Group, Nairobi, Kenya.

(*Corresponding authors: etama@strathmore.edu)

Supplementary file 4. Interview Guide – World Bank Staff

**Name**	
**Gender**	
**Job Title**	
**Qualifications**	
**Name of Organisation**	

Respondent Profile

1. How did you come to be involved with KePSIE?

Role in regulatory reforms

2. How have you been involved in the joint health inspections and the KePSIE project? (Probe - role details in design, roll out, implementation, evaluation and scale-up)

Implementation Process

3. What do you think of the Joint Health Inspections Checklist (JHIC) - What has worked well and what has not worked well and why?
    a. What do you think of having a Joint Health Inspection Checklist across all boards?

   b. What do you think about a solo inspector of any health cadre being recruited and trained to inspect a full facility?
   c. What are your views on the content of the inspections? Is it relevant to all facility levels? Probe-unfair to smaller facilities
   d. What do you think of the scoring system? (different questions in the checklist having more weight than others). Do you think the scoring system is fair?
   e. What do you think of the time between inspections and it being tied to scores?
4. What do you think of the training and selection of inspectors?
5. How significant was the mapping exercise to the implementation of KePSIE?
6. What do you think of the process of inspections in practice? (what worked well? What did not work well and why?)
7. From our data it seems there was tension between the WBCs and the inspectors. What is your perspective on this?
8. What do you think about the online monitoring system? (Was it useful? What could have been improved?)
9. What are your perceptions about the use of scorecards in facilities?
   a. What do you think about the idea of scoring and ranking facilities?
   b. What do you think about the suitability and appropriateness of the scorecard design and content? (Probe ease of comprehension and catchiness)
   c. Do you think scorecards affected facility performance?
   d. Do you think there are other better ways to communicate inspection results to the community?

Licensing and closures

10. What do you think of the licensing process for:
    a. The facility? Should this be applied to public facilities?
    b. The laboratory?
    c. The pharmacy?
    d. Individual staff?
11. What do you think of the closure of:
    a. Unlicensed facilities
    b. Unlicensed departments
    c. Should it be done?

d. Do you think it is ok to leave a registered facility operating if it scored poorly (D or C)?
e. What do you think of the process of closure?
f. How did facilities react to closures?

Factors affecting implementation

12. Have you seen any variation in KePSIE implementation across facility types (public/FBO/private), facility level (hospital/health centre/dispensary) or across counties, across inspectors?
13. Have there been deviations from the way the regulatory reforms were designed to work? What type and why?
14. Do you think implementation would have been different if it was not being evaluated through an RCT? How?
15. How significant was the role of the World Bank in the implementation? Probe-spot checks and quality checks

Governance

16. What did you think about the role of counties in implementing the JHIs? (could their roles have been different? How much buy-in was there from counties?)

Impact of reforms

17. So KePSIE had a significant effect on JHIC scores but not on IPC observation. What is your interpretation of these findings?
18. Do you think there are other benefits of KePSIE that are not capture in the evaluation results?
19. Do you think the reforms are seen as fair and legitimate by the facilities? By the counties?
20. Do you the KePSIE design succeeded in reducing the costs of inspections?
21. Would you say there were any unintended negative consequences/impacts that resulted from the implementation of the regulatory reforms? Why?
22. How do these regulatory reforms compare to the previous regulation strategy in terms of potential for bribing?

Way forward

23. What would you propose be done to improve the implementation of the project and achieve better outcomes?
24. What are your perceptions about scaling up the project to all the counties?
    a. What would be the challenges?
    b. What components of the project should be adopted and strengthened?
    c. What should be done differently and how?

d. Do you think the KePSIE model would have been scalable?

25. Is there anything else you would like to add?

Many thanks for your time – Do you have any question(s) for us?
