# Supplementary material for: What Lies Behind Successful Regulation? A Qualitative Evaluation of Pilot Implementation of Kenya’s Health Facility Inspection Reforms
Source: Int J Health Policy Manag. 2021 Aug 25;11(9):1852–62. doi: 10.34172/ijhpm.2021.90 (PMC9808232; doi:10.34172/ijhpm.2021.90)
Supplement: Supplementary file 5 — Coding Tree. [file ijhpm-11-1852-s005.pdf]

**Article title:** What Lies Behind Successful Regulation? A Qualitative Evaluation of Pilot Implementation of Kenya's Health Facility Inspection Reforms

**Journal name:** International Journal of Health Policy and Management (IJHPM)

**Authors' information:** Eric Tama[1]*, Irene Khayoni[1], Catherine Goodman[2], Dosila Ogira[1], Timothy Chege[1], Njeri Gitau[3], Francis Wafula[1]

[1]Institute of Healthcare Management, Strathmore University Business School, Strathmore University, Nairobi, Kenya.

[2]Department of Global Health and Development, London School of Hygiene and Tropical Medicine, University of London, London, UK.

[3]World Bank Group, Nairobi, Kenya.

(*Corresponding authors: etama@strathmore.edu)

**Supplementary file 5.** Coding Tree

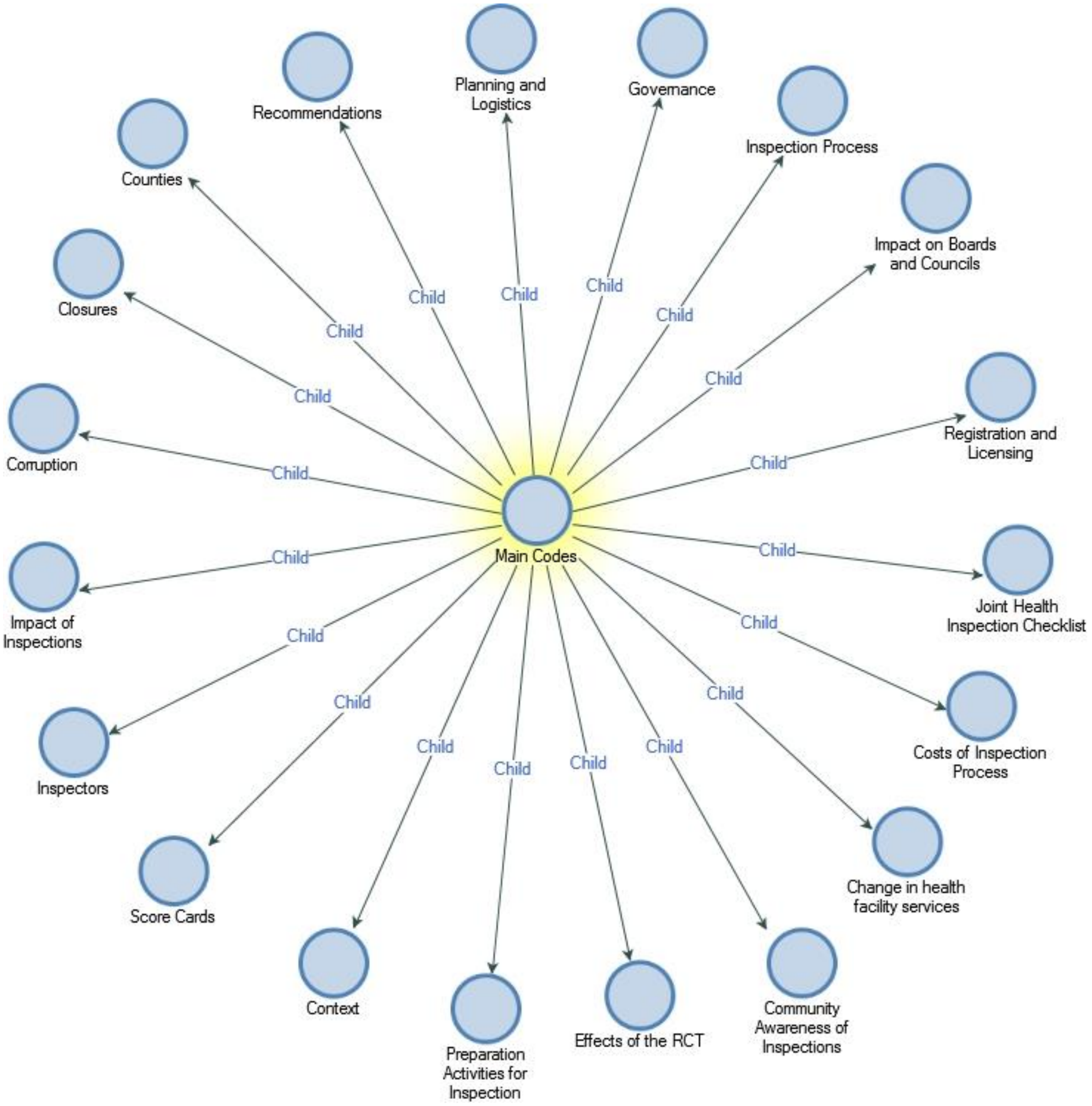
Main Codes
Planning and Logistics
Governance
Recommendations
Inspection Process
Counties
Impact on Boards and Councils
Closures
Registration and Licensing
Corruption
Joint Health Inspection Checklist
Impact of Inspections
Costs of Inspection Process
Inspectors
Change in health facility services
Score Cards
Context
Preparation Activities for Inspection
Effects of the RCT
Community Awareness of Inspections
Child
